# Habitat management interventions for a specialist mid- successional grassland butterfly, the Lulworth Skipper

**DOI:** 10.1007/s10841-024-00638-4

**Published:** 2024-11-20

**Authors:** Rachel Jones, Robert Wilson, Ilya Maclean, Nigel Bourn

**Affiliations:** 1 Environment & Sustainability Institute, University of Exeter, Penryn Campus, Cornwall, TR10 9FE UK; 2 Butterfly Conservation, Manor Yard, East Lulworth, Dorset, BH20 5QP UK; 3 Museo Nacional de Ciencias Naturales (MNCN-CSIC), Madrid, 28006 Spain

**Keywords:** Mid-successional species, Butterflies, Habitat management, Management trials, *Thymelicus acteon*

## Abstract

Evidence-based management is needed to reverse declines in insect abundance. The Lulworth Skipper *Thymelicus acteon* is a range-restricted and declining species in the UK and northern Europe associated with mid-successional grassland, which presents management challenges because interventions are necessary to prevent long-term habitat deterioration but can result in short-term reductions in quality. In addition, site management should be compatible for the focal species and for wider plant and insect diversity. We conducted factorial experimental management trials to understand effects of cutting and rotovation on the height and structure of vegetation containing the larval host plant Tor-grass *Brachypodium rupestre*. We monitored vegetation height, *B. rupestre* cover and plant diversity, and *T. acteon* larval presence over four years. Rotovation and cutting differed in their effects on habitat structure and larval occupancy relative to controls. Vegetation height and host plant cover, the most important components of habitat quality for *T. acteon*, were faster to recover to suitable levels on cut plots. However, larval occupancy increased more quickly on rotovated plots, where plant species diversity was also higher. Results suggest that due to initial negative impacts of interventions on *T. acteon* occupancy, low frequency or low-intensity management, such as managing sections of a site every three years, is advisable. Our results show that rotovation or cutting the sward can be suitable for mid-successional grassland species such as Lulworth Skipper on sites where grazing might be problematic. Rotational grazing or rotovation can maintain suitable conditions for habitat specialist insects requiring a range of different grassland conditions, serving wider conservation goals.

**Supplementary Information:**

The online version contains supplementary material available at 10.1007/s10841-024-00638-4.

## Introduction

Abundance and distribution declines of insects are well documented, with key drivers identified as habitat loss and degradation, climate change, and land use change including agricultural intensification (Warren et al. 2021; Forister et al. 2021; Outhwaite et al. 2022; Harvey et al. 2023). Managing habitat quality can help towards increasing population abundance, with implications for site and landscape-level occupancy and reducing rates of distribution decline (Ellis et al. 2011; Thomas et al. 2011; Warren et al. 2021). At higher habitat quality, sites support larger, less vulnerable populations. As a result, functional connectivity for a given configuration of the landscape is improved, as the number of potential dispersers and hence likelihood of movement between patches increase with population abundance (Thomas et al. 2001; Heisswolf et al. 2009; Hodgson et al. 2011a). Habitat quality for insects can be defined by the extent to which there are suitable resources and microclimates for development, for example, larval host plants within a particular vegetation height (Schtickzelle et al. 2007; Simons et al. 2023; Johansson et al. 2024), tall vegetation to provide shelter (Gardiner and Hassall 2009; Turner et al. 2009) or areas of open ground (Davies et al. 2006; Thomas et al. 2011). Mid-successional species such as the butterflies *Hamearis lucina*,* Carterocephalus palaemon and* some *Orthoptera* species (e.g. *Chorthippus parallelus)* require taller vegetation or light scrub (Ravenscroft 1994; Schtickzelle et al. 2007; Gardiner and Hassall 2009; Turner et al. 2009; Hayes et al. 2021; Jones et al. 2023a; Schwarz et al. 2023) but management is required to prevent an overgrowth of scrub and rank vegetation (Balmer and Erhardt 2000). However, intense or high frequency management has a detrimental effect on populations (Kruess and Tscharntke 2002; Johansson et al. 2019). Furthermore, habitat loss has exacerbated management pressures on remaining habitat fragments, by increasing the chances of management conflicts between species with different requirements. Management within sites and across landscapes often needs to maintain habitats for species using different ends of the successional scale (e.g. short and long turf) and therefore management should promote habitat heterogeneity. Habitat management for mid-successional species is therefore challenging not only in maintaining the balance between retaining longer vegetation and reversing succession to promote suitable habitats in the long term, but needs to take place alongside creation of habitats for species with other successional requirements (especially early successional plants and insects; e.g. Thomas et al. 2001; Bourn and Thomas 2002; Helbing et al. 2014).

The Lulworth Skipper *Thymelicus acteon* is an example of a mid-successional species associated with lightly grazed or ungrazed sites (Thomas 1983a; Bourn and Thomas 2002). *T. acteon* has experienced distribution declines and moderate abundance declines in Europe (Van Swaay et al. 2019), significant declines in distribution and abundance in England (Fox et al. 2023) and it is classified as Near Threatened in European (Van Swaay et al. 2010) and British (Fox et al. 2022) Red Lists. In the UK, *T. acteon* reaches its northern range edge in southern England, where it is restricted to a 40 km stretch of coastline in agriculturally unimproved habitats of south-facing calcareous grassland with a high cover of the larval host plant, Tor-grass *Brachypodium rupestre* (Thomas 1983a; Thomas et al. 2001; Jones et al. 2023a). Despite climate change and evidence of range expansions in similar species (Hill et al. 2002; Davies et al. 2005; Lawson et al. 2012; Fox et al. 2015), a lack of suitable habitats and low dispersal capacity mean there has been no evidence of an expansion in the range of *T. acteon* in the UK in the last 40 years (Jones et al. 2023a). Metapopulation modelling shows that improved habitat quality across the population network could help facilitate *T. acteon* expansion (Jones et al. 2023a) however, though there has been research on *T. acteon* habitat associations (Thomas 1983a; Bourn and Thomas 2002; Jones et al. 2023a), metapopulation dynamics (Thomas et al. 2001; Jones et al. 2023a), dispersal (Thomas 1983b; Louy et al. 2007) and phenology (Stefanescu et al. 2003; Brooks et al. 2017) there has been no specific research on habitat management for the species. Given the effects of habitat quality on population dynamics of *T. acteon* (Jones et al. 2023a) it is important to improve the understanding of how to optimise management for this species.

*Thymelicus acteon* is known to be responsive to management which results in a changed vegetation structure (Thomas 1983a; Thomas et al. 2001), and the highest densities occur where vegetation grows to intermediate vegetation heights (20–35 cm) (Jones et al. 2023a). In the UK *T. acteon* has a single annual life cycle, flying from May to August, and females lay batches of up to 15 eggs in the grass stems of the larval host plant *B. rupestre.* Upon hatching, first instar larvae overwinter at the oviposition site before feeding high up in the grass on leaves of *B. rupestre* in early spring. Frequent or intensive management has a negative effect on *T. acteon* as these early life-stages are high up in the tall grass tussocks for most of the year. However, population density is reduced in vegetation under 15 cm or over 40 cm tall (Thomas 1983a; Jones et al. 2023a), and unmanaged sites are prone to scrub encroachment which can directly reduce the habitat area and affect metapopulation dynamics (Hanski 1994; Thomas et al. 2001; Jones et al. 2023a). Though *T. acteon* is associated with ungrazed or lightly grazed habitats (Thomas 1983a; Bourn and Thomas 2002) we lack an understanding of suitable management techniques, particularly in locations where light grazing is problematic due to human abandonment (causing excessive scrub encroachment, reducing access to remaining grassland), health and safety of livestock (limited access e.g. steep slopes, cliff tops and verges) and economic issues associated with implementing viable grazing on low productivity grasslands (e.g. installing fencing or water supply on steep or remote sites). *T. acteon* also inhabits a landscape which supports a range of species with different habitat needs, many of which require intense grazing, including the short-turf species Adonis Blue *Polyommatus bellargus* (Thomas et al. 2001), increasing potential management conflicts on some ungrazed sites. Understanding how to optimise management on sites where grazing is problematic is therefore an important conservation issue.

Management trials can provide an evidence base for understanding effects of specific interventions on a target species (Ellis 2003; Korösi et al. 2014) or communities (Grill et al. 2008; Hamřík and Košulič 2021). This study tests effects of cutting and rotovation on *T. acteon* occupancy and on wider aspects of habitat in grasslands that are primarily managed for conservation and public recreation. Rotovation, whereby the ground surface and sub-surface is disturbed, breaks up dense litter, roots and rhizomes, potentially weakening *B. rupestre* and creating germination gaps for other plant species. Cutting removes scrub and rank vegetation, reducing *B. rupestre* dominance (Bobbink and Willems 1991; Bonanomi et al. 2006) bringing vegetation height back towards an optimum. The larval host, *Brachypodium rupestre*, has been a focus of other grassland management trials (Bobbink and Willems 1987, 1991, 1993; Bonanomi et al. 2006; Redhead et al. 2019) as it is a dominant species which spreads through rhizomes, is often avoided by livestock, and is associated with low plant diversity. We expect management initially to have a detrimental effect on *T. acteon* occupancy as vegetation height and host plant resource reduce to suboptimal levels (Jones et al. 2023a). We also consider wider species diversity and expect plant species diversity to temporarily increase when above-ground biomass is removed and there is ground disturbance (Bobbink and Willems 1993; Hurst and John 1999). From the results of the trials, we make recommendations for ongoing management at the local and landscape-level for *T. acteon*.

## Methods

### Management trials

**Fig. 1:**
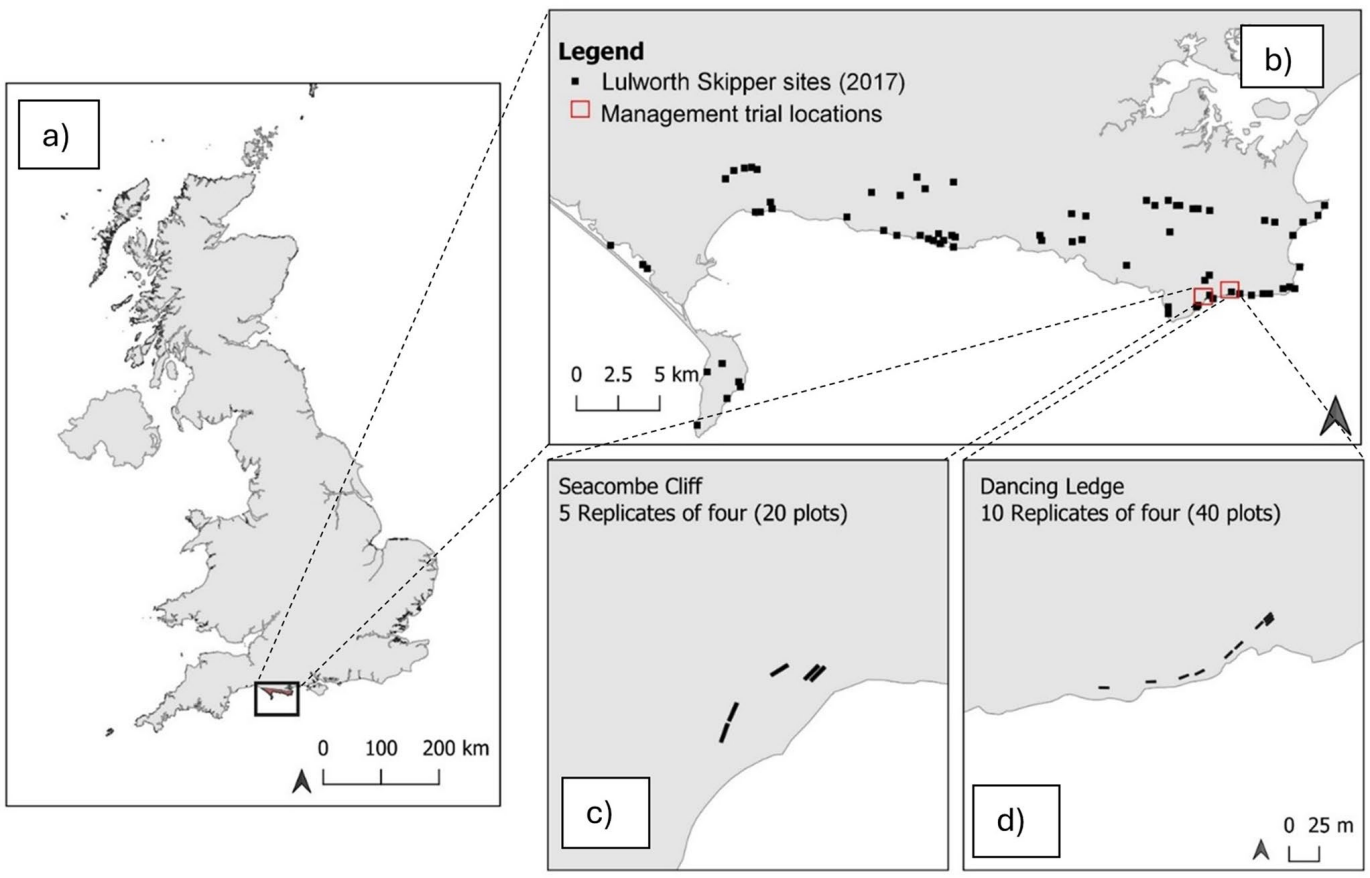
(**a**) The distribution of the Lulworth Skipper *Thymelicus acteon* in the UK; (**b**) The location of the management trial sites and occupied *T. acteon* sites in 2017 (please note a site in the far west of the distribution is not included on the map); (**c**) Location of replicates at Seacombe Cliff; d) Location of replicates at Dancing Ledge

Management trials were conducted on the south coast of Dorset, UK (Fig. 1), with two sites selected: Dancing Ledge (latitude: 50.591316, longitude: -2.0076323) and Seacombe Cliff (latitude: 50.588741, longitude: -2.0277983). The two sites are along cliff-tops and were selected as grazing is excluded by fencing, thus allowing control over effects of livestock on vegetation height and ground disturbance. Each enclosed area also included sufficient space for a number of replicate plots. The two sites were approximately 1 km apart with a south to south-easterly aspect. Both sites were similar in habitat condition (Fig S1 Supporting Information) have limestone geology and support calcareous grassland habitats dominated by *B. rupestre.* Previous work on *T. acteon* refers to *B. pinnatum* as the larval host plant. However, recent taxonomic revisions classify *B. pinnatum s.l.* as two separate taxa with *B. pinnatum* more widespread and shade tolerant and *B. rupestre* being restricted to calcareous soils (Stace 2010; Stroh et al. 2020). In England, as *T. acteon* is restricted to open calcareous grasslands, we refer to *B. rupestre* throughout as this is the more likely host species. However, distinguishing between the two plant species is difficult.

**Fig. 2:**
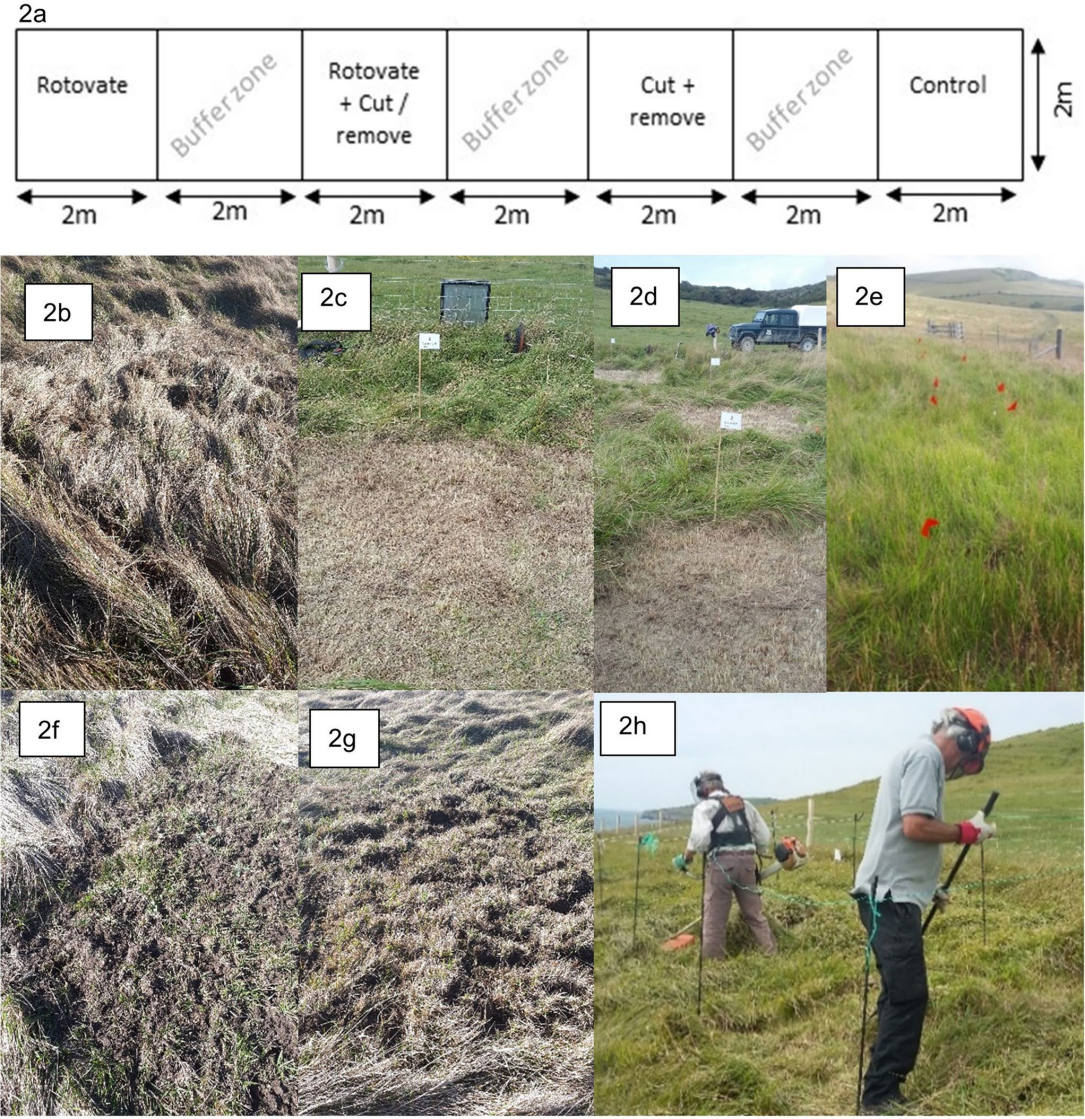
(**a**) Example of the experimental design of each replicate (treatments were allocated randomly so location of treatment varied in each plot); (**b**) rotovate (no cut) management in February 2017; (**c** and **d**) cut treatments with removal of cut material in August 2017; (**e**) Plots after three growth seasons (2020) at Seacombe Cliff, the red flag markers were used to find edges of the central 1m^2^; (**f** and **g**) Rotovate and cut / remove treatment in February 2017; (**h**) volunteers cutting plots using handheld strimmers

Fifteen replicates were set up across the two study sites, with 10 replicates at Dancing Ledge and five at Seacombe Cliff. Replicates were divided into four plots, each plot measuring 2 × 2 m with a 2 m buffer zone to account for edge effects (Fig. 2). A factorial design was used with three treatment plots and a control. Treatments were allocated randomly to the 2 × 2 m plots in each replicate to avoid bias.

#### Cut treatment

Vegetation was cut using a hand-held strimmer set to a height of 3 cm, in 30 plots (two in each replicate). All cut material was gently raked and removed from the plots. Cut treatments were conducted in August 2017 as summer cutting is more effective at reducing the dominance of perennial rhizomatous grasses such as *B. rupestre* (Bobbink and Willems 1987, 1991, 1993; Bonanomi et al. 2006). Removing grass leaves prior to senescence prevents the translocation of resources to the rhizomes for winter storage, reducing nutrients available to produce new shoots the following spring (Bobbink and Willems 1991; Kroon and Bobbink 1997). A single cut was implemented: cutting twice (spring and summer) can be more effective at temporarily increasing plant diversity, but there is little evidence of significant effects of two cuts on *B. rupestre* dominance (Bobbink and Willems 1993), and a single cut is more cost-effective for land managers.

#### Rotovation treatment

Pitchforks were used to break up the soil surface and sub-surface roots in 30 plots (two in each replicate). Hand tools were used as the existing fencing infrastructure limited access for machinery. Rotovation was conducted in October 2017 and repeated in February 2018 when ground conditions were damp enough to allow rotovation. The second treatment was implemented to ensure broken ground for Spring plant germination.

#### Rotovation and cut treatment

In one cut plot per replicate (August 2017) rotovation was also implemented in October 2017 and February 2018.

##### Control

One of the four plots per replicate was left with no cutting or rotovation.

### Data collection

Data were collected from the central 1 m^2^ of each trial plot to minimise edge effects from the unmanaged buffer zone and disturbance to the larvae during data collection in the untrampled central plot. Data on larval occupancy, vegetation height and *B. rupestre* cover were collected before the interventions (2017), then annually for four years following interventions (2018, 2019, 2020, 2021) with data on plant diversity collected in 2019 and 2021 (Table 1). *Thymelicus acteon* occupancy data were the presence or absence of larvae from a plot, with each blade of *B. rupestre* within 1 m^2^ survey plots searched for larvae. When larval presence was confirmed, the search continued, however as female *T. acteon* can lay up to 15 eggs it is not possible to ascertain whether multiple larvae were a result of oviposition preference of a single or multiple females, therefore presence rather than abundance was used in analysis. The larval searches were conducted once annually, with timing of the surveys based on the size of larvae in checks beforehand. Larval surveys were conducted in late May / early June when larvae were larger and more easily detected, minimising the risk of false absences.

Vegetation height and host plant cover are key components of habitat for *T. acteon* (Thomas 1983a; Jones et al. 2023a) and data on vegetation height (cm) and percentage cover of the host plant were collected to understand how habitat differed between plots, and how this might affect larval occupancy. Vegetation height was measured using the direct method (Stewart et al. 2002), with a 1 m rule used to estimate the level below which approximately 80% of the vegetation was growing. To capture variation in vegetation height, five measurements were taken, one in each corner (approximately 25 cm diagonally inwards from each corner) and one in the centre of each plot. Vegetation data were used to generate a mean vegetation height and coefficient of variation (vegetation structure) for each plot. Vegetation height was measured at the same time as larval surveys (May / early June) so it could be linked to larval occupancy, and measured again in July / August to coincide with the adult flight season. The percentage cover of *B. rupestre* was estimated for the 1 m^2^ search area, also at the same time as larval surveys. Plant species diversity was measured to help understand effects of reversing habitat succession on wider diversity. Each plant species within the 1 m^2^ area of each plot was identified (where possible), counted and converted into an index of plant diversity based on Simpson’s Index (Mouillot and Leprêtre 1999). Plant surveys were conducted in June 2019 and 2021 (2020 surveys were affected by COVID-19 restrictions) the timing aimed to help maximise the number of flowering plants that were visible.

### Statistical analysis

Differences between treatments in mean vegetation height (spring and summer), vegetation structure (coefficient of variation), *B. rupestre* cover and plant species diversity were assessed using Kruskal Wallis tests on data from each year post intervention (2018–2021) and post hoc pairwise Wilcoxon tests conducted within years (Bonferroni corrected for multiple comparisons) to compare differences between treatment plots. Significant differences between the habitat attributes at the two management sites, Dancing Ledge and Seacombe Cliff, were also assessed using Wilcoxon tests.

Factors affecting larval occupancy were analysed using a Generalized Linear Mixed Model (GLMM) with a binomial error structure and *logit* link function using the lme4 package (Bates et al. 2015) in RStudio ver. 1.2.1335. The first model used larval occupancy (1 = occupied, 0 = unoccupied) as the dependent variable with treatment and year since intervention as explanatory variables. As the experimental trials were set up using a factorial design, the data for treatment were structured using binary codes for cut (1 = cut, 0 = no cut) and rotovate (1 = rotovate, 0 = not rotovated), thus control plots would be coded 0, 0 and cut and rotovated plots coded 1, 1. Year since intervention excluded the 2018 data as there had been no growth season or egg-laying period since the treatments, and would not reflect *T. acteon* larval occupancy following egg-laying after the treatment. Years 2019–2021 were included in the model as a numeric variable of 2 (2019), 3 (2020) or 4 (2021) years since intervention (2019 = two growth seasons, 2020 = three growth seasons and 2021 = four growth seasons). To control for repeated measures from the plots over time, plot was included in the model as a random effect.

The effects of habitat on larval occupancy were also analysed using a GLMM with a binomial error structure and *logit* link function. Plot was included as a random effect, with larval occupancy (1 = occupied, 0 = unoccupied) again as the dependent variable. Average spring vegetation height, the quadratic effect of vegetation height, spring host plant cover, treatment and time were explanatory variables. Vegetation height data in this habitat model was scaled by predictor to standardise the data and improve model stability and convergence (Harrison et al. 2018). Treatment and time were included in the model in the same format as the first model.

The relationship between treatment and plant species diversity was also tested using a GLMM with a Gaussian error structure. Plant species diversity (Simpson’s Index) was the dependent variable, treatment and time were explanatory variables and plot a random effect. The same data format for treatment and time was used as in previous models. Assumptions for all models were checked using the DHARMa package (Hartig 2021). The MuMin package (Barton 2019) was used to compare AICc values and model outputs from a set of models decided a priori. Model averaged coefficients were generated from models ΔAICc ≤ 2 of the top model (Richards et al. 2011) but where there was no other model within two AICc of the top model then this single model was selected.

## Results

### Differences in habitat by treatment

**Fig. 3:**
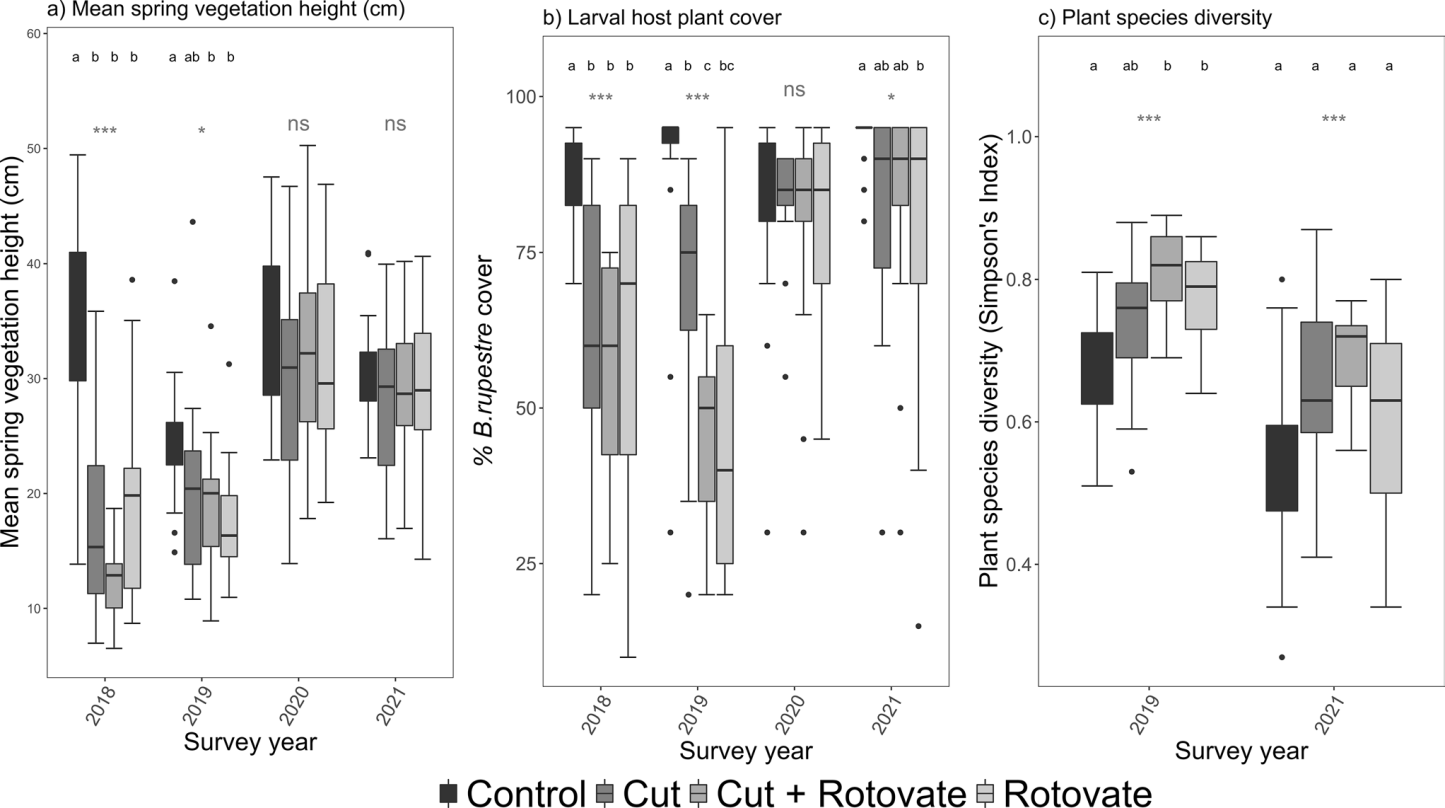
Boxplots showing; (**a**) Mean Spring vegetation height (cm) by treatment and year; (**b**) *Brachypodium rupestre* cover (%) by treatment and year; (**c**) Plant species diversity (mean Simpson’s Index) by treatment and year. Year includes the years following the interventions; 2018 (no full flight period or growth season since management), 2019 (one growth season / flight period), 2020 (two growth seasons / flight periods) and 2021 (three growth seasons / flight periods). Boxplots show the median value (horizontal line), upper and lower quartiles (box), the minimum and maximum values (whiskers) and outliers (asterisks). Significance values from Kruskal Wallis tests are indicated by *** p = < 0.0001, **p = < 0.001, *=<0.05, ns = not significant. For years with significant results the compact lettering shows results of post hoc Wilcoxon pairwise tests (p = < 0.05, Bonferroni corrected for multiple comparisons within years) with means not sharing any letter being significantly different

Average spring vegetation height differed by treatment in 2018 (p = < 0.0001, chi-squared = 26.538) and 2019 (p = < 0.005, chi-squared = 12.634). In 2018 the control treatment had significantly taller vegetation than all other treatments and in 2019 was significantly taller than plots with a rotovation treatment (post hoc Wilcoxon pairwise tests, Bonferroni corrected for multiple comparisons within years) (Fig. 3, Table S2 in Supporting Information). From 2020 (two full years after the interventions) there was no significant difference in vegetation height by treatment. *B rupestre* cover differed significantly between treatment plots in 2018 (p = < 0.0001, chi-squared = 21.288) and 2019 (p = < 0.0001, chi-squared = 26.629). In 2018 and 2019 *B. rupestre* was significantly higher in control plots than all other treatments and in 2019 *B. rupestre* cover was also significantly higher on cut plots compared to cut with rotovation (post hoc Wilcoxon pairwise tests Bonferroni corrected for multiple comparisons within years) (Fig. 3, Table S2 in Supporting Information). Plant species diversity differed significantly by treatment in both 2019 (p = < 0.0001, chi-squared = 19.869) and 2021 (p = < 0.05, chi-squared = 8.1818) with plant species diversity significantly lower in control plots than rotovation plots in 2019 (post hoc Wilcoxon pairwise tests, *P* > 0.05, Bonferroni corrected for multiple comparisons within years). In 2021 post hoc tests showed no significant difference different between treatment plots at the *p* > 0.05 (Fig. 3, Table S2 Supporting Information). After 2018 there was no significant difference between average summer vegetation height or spring vegetation structure (coefficient of variation) by treatment (Supporting Information S3). Significant differences in habitat attributes were found between sites in some years with differences in spring vegetation height (in 2020) as vegetation at Dancing Ledge was taller on average and *B. rupestre* cover in 2018 was significantly higher at Dancing Ledge compared with Seacombe Cliff (Supporting Information S4).

### Effect of treatment on larval occupancy

There were 141 larval presence records from the 300 plot searches from 2017 (pre intervention) to 2021. The 96 observations from 2019 to 2021 were used in larval occupancy GLMMs. The number of occupied plots varied between treatment, site and year (Table 1, Supporting Information S5). Prior to interventions, larval occupancy was highest in control plots, and occupancy in control plots ranged from a low of seven occupied plots (of 15) in 2019 and 2020 to a high of 14 in 2021. The variation in occupancy occurred alongside wider annual variation in *T. acteon* abundance as observed on UK Butterfly Monitoring Scheme transects (Supporting Information S6). The number of occupied treatment plots was lower than the control in the first year following interventions (Table 1) but by 2019 the rotovated plots had comparable occupancy to controls despite supporting shorter vegetation and lower *B. rupestre* cover (Fig. 3). Occupancy in cut only plots was slower to recover, but comparable to control plots by 2020.

**Table 1:** Number of plots with *Thymelicus acteon* present by treatment (*n* = 15 per treatment and control) and year (*n* = 60 plots per year)

Treatments	Thymelicus acteon Occupancy				
	2017*	2018**	2019	2020	2021
Control (*n* = 15)	10	10	7	7	14
Cut (*n* = 15)	4	1	0	7	11
Cut + Rotovate (*n* = 15)	5	1	7	9	11
Rotovate (*n* = 15)	8	6	7	6	10
**Total occupied plots** (***n* = 60)**	**27**	**18**	**21**	**29**	**46**

* prior to management interventions; ** year after interventions (no growth or flight season since management)

**Fig. 4:**
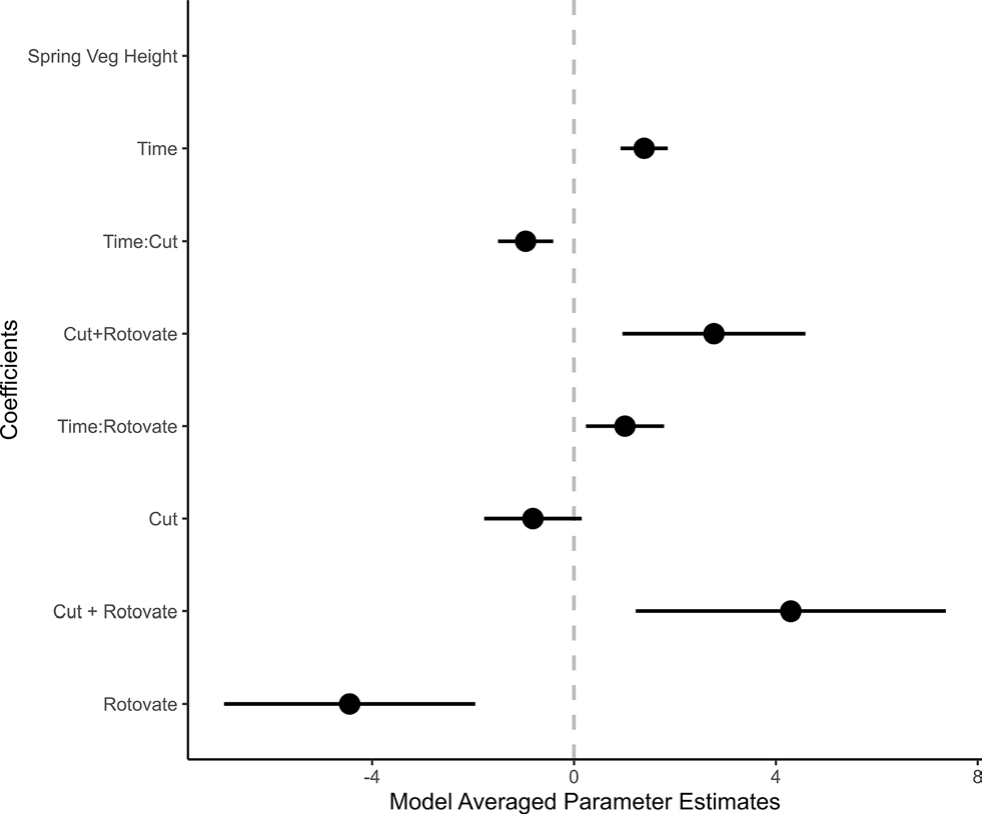
Forest plot showing model averaged parameter estimates (Models ΔAIC ≤ 2; Table 2) for effects of treatment, time and the interactions of treatment with time on larval occupancy. The bars illustrate the standard errors of parameter estimates. Apart from the three-way interaction, no parameter estimates overlap with zero (dashed grey vertical reference line)

Estimates of the expected probability of larval occupancy by treatment were derived by model averaging (Table 2). The model averaged coefficients show effects of treatment and how this effect on the probability of larval occupancy changes over time (Fig. 4). Rotovation had a positive effect on occupancy, compared to non-rotovated cut plots, but this effect decreased over time. Cutting had a negative effect on occupancy compared to uncut plots, but occupancy on cut plots was positively influenced by time and rotovation treatment.

**Fig. 5:**
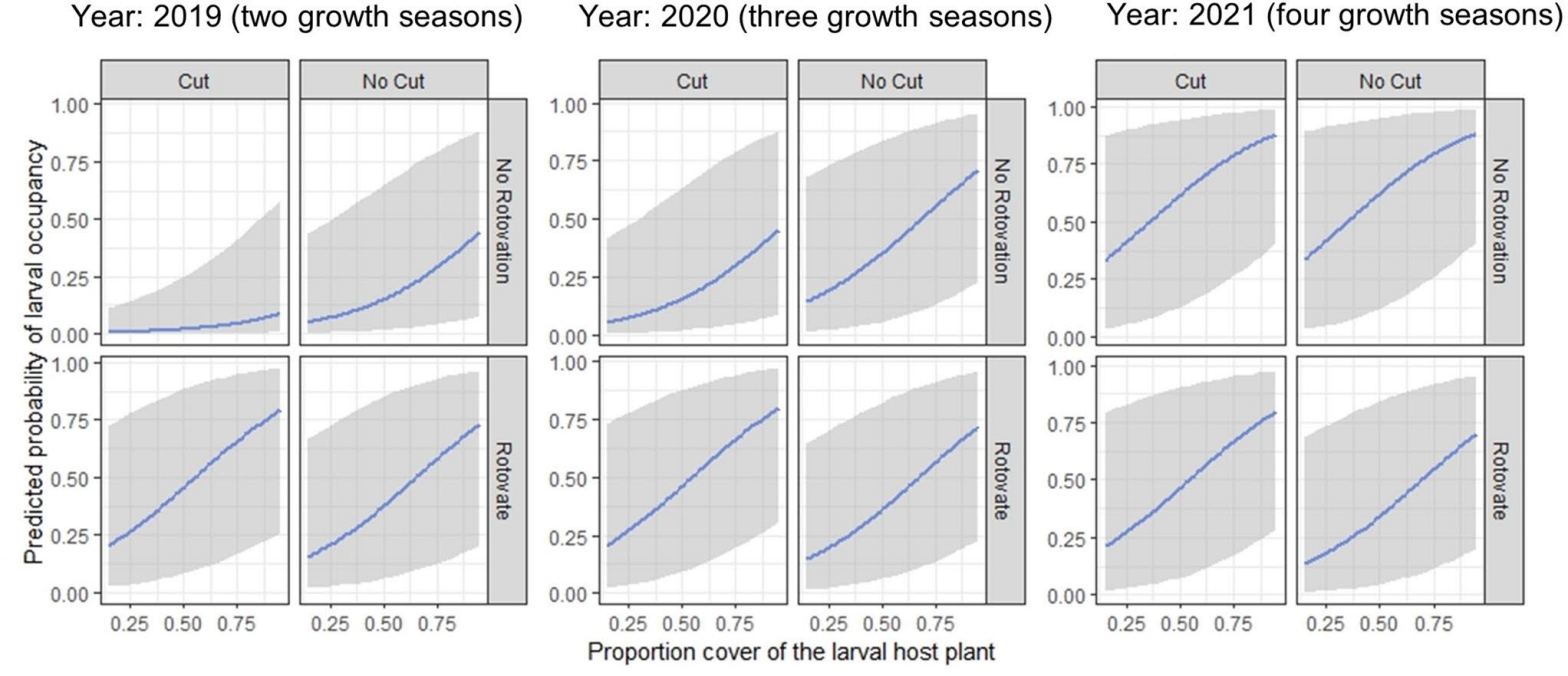
Predicted probabilities of *Thymelicus acteon* larval occupancy by proportion cover of *B. rupestre* by cut and rotovation treatment and year. Model coefficients are estimated from the most parsimonious Generalised Linear Mixed Model analysing the relationship between habitat, treatment and time on larval occupancy. Grey shading represents the upper and lower confidence intervals

The top-ranking model describing the relationship between habitat, treatment and time on larval occupancy includes effects of treatment, time and *B. rupestre* cover. *B. rupestre* cover had a positive effect on larval occupancy (Supporting Information S7) but interacted with the effect of treatment (Fig. 5), with occupancy at rotovated sites increasing with *B. rupestre* cover. No other models were within 2 ΔAICc of this model; vegetation height was included in the next ranked model (ΔAIC > 2) and had a positive effect on occupancy, particularly in cut plots (Supporting information S7).

**Table 2:** Multi-model inference of relationships between larval occupancy, treatment and time. The top three models with ∆AICc < 2 are used to derive model averaged coefficients

Model	AICc	∆AICc	Wi	Intercept	Cut	Rotovate	Cut x rotovate	Time	Cut x time	Rotovate x Time	Cut x Rotovate x Time
Model 5	229.1	0	0.467	-4.826 (1.195)		3.369 (1.455)		1.627 (0.388)		-1.055 (0.466)	
Model 8	230.2	1.1	0.269	-3.083 (1.427)	-4.445 (2.471)	1.739 (1.880)	4.297 (3.051)	1.254 (0.486)	1.010 (0.769)	-0.789 (0.620)	-0.814 (0.960)
Model 7	230.8	1.68	0.202	-2.908 (0.730)				1.027 (0.239)			
Model 4	233.2	4.06	0.061	-2.113 (0.943)	-1.683 (1.366)			0.815 (0.308)	0.450 (0.438)		
Null	252.3	23.23	0	0.140 (0.164)							
Model 6	253.4	24.29	0	0.512 (0.324)	-0.927 (0.450)	-0.466 (0.450)	1.297 (0.643)				
Model 1	253.7	24.56	0	0.280 (0.232)	-0.280 (0.326)						
Model 2	254.1	24.97	0	0.047 (0.232)		0.187 (0.326)					
Model 3	255.2	26.32	0	0.187 (0.281)	-0.280 (0.325)	0.187 (0.325)					

Note: AICc is the Akaike Information Criterion (corrected for small sample size), ∆AICc the difference in AICc from the best model and Wi is the Akaike weight. The direction of the effect is indicated by model coefficients, with standard errors shown in brackets. Models were fitted using a binomial Generalised Linear Mixed Model with plot as a random intercept

### Effect of treatment on plant diversity

**Fig. 6:**
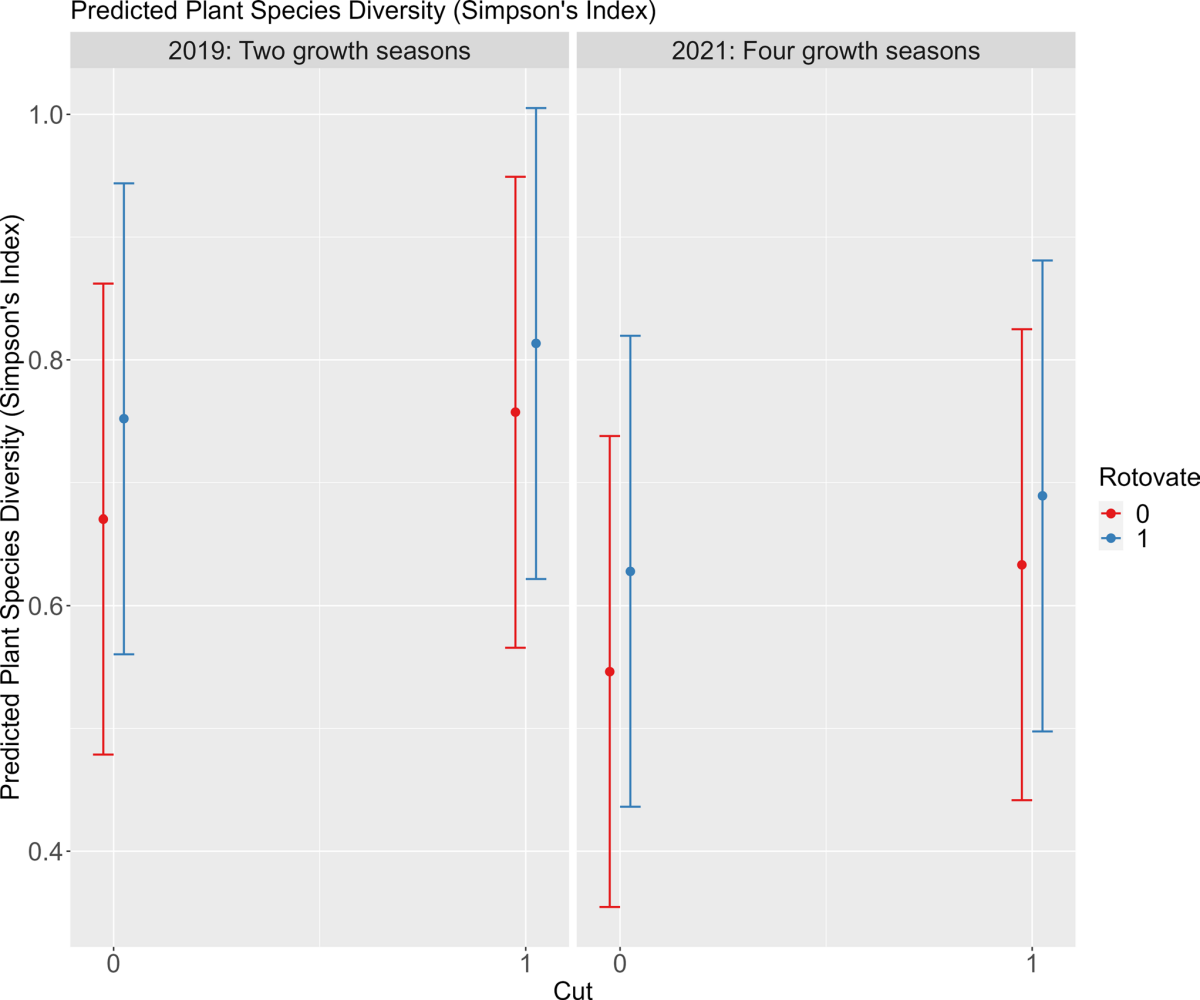
Plot of predicted plant species diversity (Simpson’s Index) for cut and rotovated treatments in 2019 (two growth seasons since intervention) and 2021 (four years since interventions). The outputs are based on the most parsimonious describing the relationship between treatment and time on occupancy with bars representing the standard errors

The most parsimonious model describing the relationship between plant species diversity and treatment was ΔAIC > 2 from the next ranked model. This model shows a positive effect of management, with a larger effect of rotovation on plant diversity compared to control or cut only plots (Fig. 6, Supporting Information S8 and S8) but the positive effect of rotovation reduces with time since intervention.

## Discussion

Improving our understanding on how to manage habitats for *Thymelicus acteon* is key to ensure suitable habitat is maintained across the population network, improving metapopulation viability and potentially to help facilitate future range expansions (Thomas et al. 2001; Jones et al. 2023a). Management trials represent an important tool in developing this understanding as the effects of specific interventions on target species (Ellis 2003; Korösi et al. 2014) or communities (Grill et al. 2008; Hamřík and Košulič 2021) can act as an evidence base to inform ongoing management. Using a factorial design with rotovation (autumn and repeated in late winter) and late summer cutting (with removal of cut material), we tested the effects of management using *T. acteon* larval occupancy as a response variable. We aimed to understand how *T. acteon* responds to management that aims to bring habitat conditions towards optimum, but which result in a lowered habitat quality in the short-term. We summarise the results, discuss management applications in the context of local and landscape management for *T. acteon* and plant diversity, then outline broader implications.

### Response of *Thymelicus acteon* to management

*Thymelicus acteon* is responsive to management which results in a changed vegetation structure (Thomas 1983a; Thomas et al. 2001) with population densities highest in mid-length swards between 20 and 35 cm tall and lowest in short (< 15 cm) or tall (> 40 cm) vegetation (Jones et al. 2023a). Cutting and rotovation treatments altered the vegetation structure and cover of the host plant, through the physical removal of above-ground biomass (cutting and raking), or by breaking up roots and rhizomes (rotovation). Negative effects on *T. acteon* occupancy in the first season following interventions are expected owing to sudden changes in habitat structure, reductions in host plant availability and (in cut plots) the removal of overwintering larvae. We found that larval presence following interventions was reduced for the first year compared with controls in plots with rotovation treatments, but for two years in cut plots. No larvae were recorded in cut plots in 2019, two growth seasons following management. Over time, occupancy improved in treatment plots and was at a similar level in all treatments as the control in 2020, three growth seasons after interventions. There was no evidence for an improvement in occupancy in treatment plots, relative to the control, however, habitat conditions in the control plots were on average still within suitable bounds for *T. acteon* (15–40 cm vegetation height and > 30% *B. rupestre* cover, Jones et al. 2023a) and starting trials in sub-optimum habitat across treatment plots may have shown improvements in the managed plots relative to control plots.

Understanding how long it takes for population recovery following management can guide frequency or intensity of future interventions. *T. acteon* recovered on rotovated plots by 2019 after a single flight season, indicating the potential presence of suitable conditions for oviposition as soon as the first summer afterwards. Cut only plots had a slower recovery, with no cut plots occupied in 2019, despite a more rapid recovery in vegetation height and *B. rupestre* cover. Combining cutting with rotovation resulted in a quicker recovery than cut only treatments, possibly due to vegetation structure, as cutting alone can leave a uniform vegetation (Tälle et al. 2016), however coefficient of variation in vegetation measurements (the measure of vegetation structure) was not significantly different between treatments. Rotovation had effects on occupancy, as host plant cover was reduced, but occupancy recovered rapidly and was comparable to the control by 2019. The response of larval occupancy to management interventions shows that rotovation and cutting are both useful management tools as effects on occupancy were temporary and populations had recovered three growth seasons following interventions.

Understanding the effects of an intervention on habitat quality and recovery to suitable conditions helps improve understanding of the factors which determine the success of management (Ellis 2003; Grill et al. 2008; Gardiner and Hassall 2009). Vegetation height in treatment plots was shorter and *B. rupestre* cover lower than the control in the first two surveys following interventions; however, despite a reduction they remained above lower niche thresholds for *T. acteon* (15 cm and 30% respectively). Larval occupancy was more sensitive to availability of the host plant (see also Thomas et al. 2011) and overall rotovated plots with high *B. rupestre* cover had higher probability of occupancy than cut plots or rotovated plots with low *B. rupestre* cover. Recovery of *B. rupestre* cover to levels similar to controls was faster on cut plots, possibly due to effects of rotovation on sub-surface rhizomes and the creation of germination gaps for the growth of other species that could temporarily suppress *B. rupestre* growth. The rapid recovery of habitat and occupancy in the treatment plots to levels similar to controls suggests that either cutting or rotovation are useful management options where vegetation height risks exceeding suitable thresholds for the species. However, maintaining a high cover of mid-height *B. rupestre* in and around management locations as refuges (Scherer and Fartmann 2023) could help to buffer *T. acteon* from any initial adverse effects of management and promote source populations for recolonisation.

### Treatment effects on plant species diversity

Habitat loss and fragmentation due to abandonment and afforestation and agricultural intensification have reduced the availability of semi-natural grasslands (Ridding et al. 2020) and remaining fragments often support species with conflicting needs which present a management challenge. *B. rupestre* is a competitive species that forms dense litter layers which suppress plant growth. Due to negative effects on diversity, research on management to reduce its dominance is ongoing (Bobbink et al. 1987; Bobbink and Willems 1991; Buckland et al. 2001; Redhead et al. 2019), although rotovation has rarely been tested. Optimum habitat for *T. acteon* (high *B. rupestre* cover and mid-height vegetation) results in a trade off with habitats with a higher diversity of plants. However, managing habitat for mid-successional species like *T. acteon* can temporarily reverse succession, providing opportunities for other plant species. Increasing plant diversity has benefits associated with heterogeneity in microclimate, food and nectar. Though maintaining a high dominance of *B. rupestre* is important for *T. acteon*, it is beneficial to have an understanding of how management interventions might maximise plant diversity.

Short-term increases in plant diversity were observed in treatment plots compared to controls, particularly where rotovation and cutting were combined. Rotovation and cutting could be effective by combining ground disturbance with removal of above ground biomass (litter and growth). Cutting and removal of vegetation (including the litter) allows more light to the ground, affecting microclimates and encouraging growth of plant species (Bobbink and Willems 1987, 1991), and rotovation creates germination gaps (Kiss et al. 2021). The additional species included single or occasional records of plants associated with calcareous habitats (Fairy Flax *Linum catharticum*, Ploughman’s Spikenard *Inula conyzae*, Bee Orchid *Ophrys apifera)*, to more widespread species (Wild Carrot *Daucus carota*, Yarrow *Achillea millefolium*, Agrimony *Agrimonia eupatoria*, Common Bird’s-foot-trefoil *Lotus corniculatus*), grasses (Cock’s-foot *Dactylus glomerata*, Fescues *Festuca spp* and False oat-grass *Arrhenatherum elatius*) and more ruderal species or those associated with disturbed ground (Smooth Sow-thistle *Sonchus oleraceus*, Ragwort *Senecio jacobaea*, Spear Thistle *Cirsium vulgare*). Many of these species act as nectar plants for butterflies including *T. acteon*, or as larval host plants for other butterflies occurring in the same grasslands. Plant diversity decreased over time as *B. rupestre* increased in dominance, with the increase in plant diversity associated with the short-term reduction in *B. rupestre* cover.

The additional benefits for wider grassland diversity of managing habitats are only short-term, as *B. rupestre* dominance increases and other plants are shaded out. Single cuts of *B. rupestre* are documented to have limited effectiveness, and are unlikely to increase plant diversity in the long-term (Redhead et al. 2019). Once *B. rupestre* exceeds 50% cover it begins to influence plant communities (Bobbink and Willems 1987), which is likely why rotovation is more effective as *B. rupestre* cover is reduced for a longer period. Repeated annual management might be required to increase plant species diversity in the longer-term as dominance of *B. rupestre* would reduce (Bobbink and Willems 1991, 1993) but in our study system this could have adverse impacts on *T. acteon.* The aim of our trials was to assess effects of management on *T. acteon* occupancy in restraining excessive vegetation growth that would be negative for this and other grassland specialists in the long term. However, the short-term benefits on plant species diversity, particularly in cut and rotovated plots, adds value to management and rotational management over space may also benefit plant diversity.

### Habitat management for *Thymelicus acteon*

Our results have practical applications particularly in locations where grazing might be problematic due to access (limiting installations of troughs or fencing), economics of grazing low productivity grasslands (resulting in abandonment) and the health and safety of livestock (e.g. habitats on cliff-tops or verges). Such sites are at risk of scrub encroachment or grass growth beyond suitable thresholds. Results support the use of light intensity or low frequency management for species such as *T. acteon* and others sensitive to intensive management (e.g. Schtickzelle et al. 2007; Humbert et al. 2012; Johansson et al. 2019). Low frequency or light intensity management helps promote overwintering survival in patches of taller unmanaged grass refuges (Scherer and Fartmann 2023; Schwarz et al. 2023), facilitating recolonisation of managed areas by species with a limited dispersal capacity such as *T. acteon* (Thomas 1983a; Thomas et al. 2001). A management regime whereby one third of habitat is managed, and two, but preferably three growth seasons allowed between rotations to enable species recovery, is proposed as a suitable strategy for managing *T. acteon* habitats where practical and feasible.

Cutting or rotovation (or a combination of both) represent viable options where management is targeted towards *T. acteon* and where grazing regimes are not practical to implement. Rotovation (no cut) had reduced impacts on initial *T. acteon* occupancy compared to cutting treatments and therefore might be the preferable option, particularly on small or isolated sites where there is limited scope to maintain areas of suitable habitat to support populations for recolonisation (e.g. small sites where most of the habitat is beyond upper vegetation thresholds). Cutting however, might be preferable on steeper terrain where management is logistically more difficult, or in larger habitat areas as it is less labour intensive than rotovating and may have benefits for farmers who could use cut material for hay. Rotovation had a positive effect on occupancy on cut plots, but combining cutting with rotovation did not have additional benefits for *T. acteon* compared to rotovating without cutting (although plant species diversity was higher where the two management techniques were combined). On larger grazed sites, though not tested, results support existing recommendations of light intensity grazing using heavy herbivores such as cattle (Thomas 1983a; Jones et al. 2023a), as cattle grazing results in the patchy removal of above ground biomass and poaching to create germination gaps (similar to cutting and rotovation effects).

For mid-successional species, implementing management rotations at a site and landscape-level so sites are not all managed simultaneously helps to maintain high-quality habitats over space and time to promote population continuity. The habitat network for *T. acteon* is generally characterised by larger grazed sites alongside unmanaged and often smaller areas of habitat where grazing is problematic or unviable (steep slopes, verges, coastal fringes and quarry sites). Within the landscape for *T. acteon* there are also sites at the successional extremes, including sites where even cutting and rotovation are not practical, including unstable cliff-edges, landslips or steep slopes within sites, which would be difficult or costly to cut or rotovate. These sites are likely to become scrub-dominated without management unless natural processes, such as landslips, reverse succession, however, on these very steep sites with shallow soils the succession rates may be slower and therefore these sites may require less frequent management. The landscape also contains sites managed for species requiring early successional stages (e.g. *Polyommatus bellargus* and *Ophrys sphegodes*) (Thomas et al. 2001), these management conflicts mean that suitable conditions for *T. acteon* might not be maintained. Ungrazed (but manageable) habitats can therefore act as refuges for *T. acteon* alongside more heavily grazed and late successional sites. Without rotational management of ungrazed habitats there is a risk habitat degradation and loss to scrub succession which could affect metapopulation dynamics and future range expansion of *T. acteon* (Jones et al. 2023a). Optimising habitats on smaller sites increases the population density and functional connectivity in the *T. acteon* metapopulation (Jones et al. 2023a) enabling small sites to act as stepping-stones in range recovery (Hodgson et al. 2011b; Kuussaari et al. 2015; Poniatowski et al. 2018). Furthermore, as many unmanaged sites link with grazed areas they can help increase local microclimatic heterogeneity and act as refuges in years of climate extremes (heatwaves and drought), potentially offering cooler microclimates to buffer populations of *T. acteon* and other species on grazed sites (Kindvall 1995; Ashton et al. 2009; Rytteri et al. 2021). Optimising rotational habitat management across population networks to balance suitable habitats with sub-optimal habitat patches at different ends of the successional scale can help maximise habitat quality, promote stable metapopulation dynamics and potentially facilitate expansion (Bulman et al. 2007; Johansson et al. 2017; Jones et al. 2023a).

### Broader applications and further research

Our results demonstrate habitat management options for a declining grassland species, particularly for sites where grazing is not possible or feasible. However, there is scope for further work to optimise current and future habitat management for *T. acteon*. Firstly, though we can suggest low frequency or intensity management, the effects of intensity or frequency were not tested. Repeated cutting is associated with reduced *B. rupestre* cover (Bobbink and Willems 1987, 1991, 1993) and though this might have benefits for wider species diversity, it would likely reduce habitat quality and therefore population densities of *T. acteon*. Furthermore, repeated cutting can also result in a loss of habitat heterogeneity (Tälle et al. 2016), but it is unclear how this might affect *T. acteon* and wider species diversity. Secondly, the seasonality of management was not tested, for some mid-successional species there may be scope to target management to times of the year when the species is less vulnerable (Johansson et al. 2024). As *T. acteon* utilises tall *B. rupestre* for most of the year (with the exception of the pupal stage which can overlap with larvae or ovipositing females) management at any time could directly impact the species. However, timing of management might affect resulting habitat differently; cutting following host plant senescence may allow *B. rupestre* to translocate resources to the rhizomes for winter storage and its dominance might be less impacted, or cutting during the growth season could have greater impacts on the recovery time of *T. acteon* occupancy. Further management trials could help optimise management recommendations for *T. acteon* and other mid-successional species to which similar lessons apply.

Habitat suitability can be affected by external drivers such as nitrogen deposition and climate change (WallisDeVries and van Swaay 2006; Forister et al. 2019; Warren et al. 2021) and understanding effects of these wider drivers on habitat quality and management is important to help maintain resilient populations. Climate change can affect plant communities (Bennie et al. 2006), larval hostplant condition (e.g. during extreme events) (Piessens et al. 2009; Johansson et al. 2020) and the breadth of habitats a species can use (Davies et al. 2006; Bennie et al. 2013). In the context of our study system climate change might affect the lower and upper threshold limits of suitable vegetation height and therefore the intensity of management *T. acteon* could tolerate (e.g. if short vegetation becomes too drought-prone or exceeds the upper thermal tolerance), or threshold turf heights above which unmanaged sites become unsuitable, but there are likely complex interactions with effects of other drivers (e.g. nitrogen deposition, increasing rainfall). Ensuring microclimatic variation at the site and landscape level can help *T. acteon* buffer effects of climate change (Suggitt et al. 2018; Jones et al. 2023b). Understanding how external drivers impact habitat suitability will be important in the development of future management techniques and in climate adaptation (Maalouf et al. 2012; Greenwood et al. 2016) and could be developed as a focus of future research to ensure that management techniques continue to promote suitable habitats.

### Implications for conservation management

Experimental trials aim to provide an evidence base to inform habitat management to support populations for focal species (Ellis 2003; Korösi et al. 2014) or wider diversity (Gardiner and Hassall 2009). We show that *T. acteon* larval occupancy recovers in managed compared to control plots after two full growth and butterfly flight seasons (three for plots which are cut only). All treatments help increase overall plant diversity in the short-term, but plant diversity is higher in treatments which include rotovation, which also had less negative effect on larvae in the first years following interventions. Rotovation therefore offers an option for management on ungrazed sites where the aim is to restore habitats for *T. acteon* and maximise short-term plant diversity. Though we did not find an improvement in *T. acteon* occupancy relative to control plots, the risk of succession to scrub is a gradual, longer-term change and unlikely to occur over the four years of our study. Our results provide lessons for habitat management on sites where grazing is not easy to implement, and where cutting or rotovation might be feasible, including evidence of short-term benefits to plant diversity. Evidence for longer term effects of interventions on the diversity of grassland plants and arthropods, and on the abundance of *T. acteon*, will require continued monitoring of habitat and insect taxa on sites subject to conservation management.

## Electronic supplementary material

Below is the link to the electronic supplementary material.

Supplementary Material 1

## Data Availability

The data that support the findings of this study are available for download from Dryad (DOI: 10.5061/dryad.mgqnk996v).
