# Supplementary material for: Habitat management interventions for a specialist mid- successional grassland butterfly, the Lulworth Skipper
Source: J Insect Conserv. 2024 Nov 20;29(1):2. doi: 10.1007/s10841-024-00638-4 (PMC11870902; doi:10.1007/s10841-024-00638-4)
Supplement: Supplementary file 1 — Supplementary Material 1 [file 10841_2024_638_MOESM1_ESM.docx]

**Supplementary Information**

Journal of Insect Conservation

Habitat management interventions for a specialist mid-successional grassland butterfly, the Lulworth Skipper

Rachel Jones ^1,2^, Robert Wilson ^3^, Ilya Maclean ^1^ and Nigel Bourn ^2^

^1^ Environment & Sustainability Institute, University of Exeter, Penryn Campus, Cornwall TR10 9FE, UK

^2^ Butterfly Conservation, Manor Yard, East Lulworth, Dorset, BH20 5QP, UK

^3^ Museo Nacional de Ciencias Naturales (MNCN-CSIC), Madrid 28006, Spain

^*^Corresponding author: [rj327@exeter.ac.uk](mailto:rj327@exeter.ac.uk)

**Figure S1:** Spring vegetation height (cm) (left) and *Brachypodium rupestre* frequency (right) in 2017 before management interventions were undertaken. Boxplots show the median value (horizontal line), upper and lower quartiles (box), the minimum and maximum values (whiskers) and outliers (open circles). Results from Kruskal Wallis tests showed no significant differences in the habitat values between treatment type prior to management taking place.

**
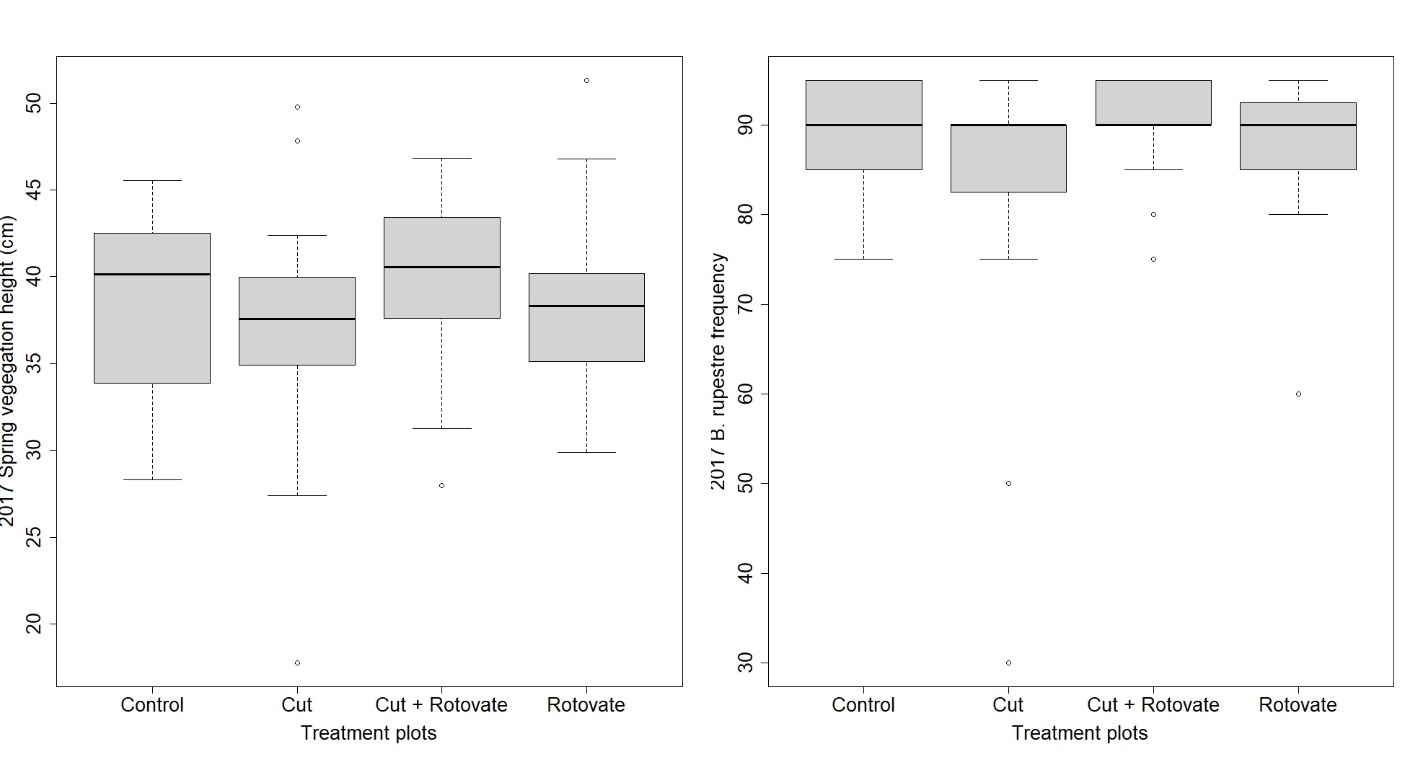
**

**Table S2**: Results from post hoc Wilcoxon pairwise tests for significant differences in habitat values between treatments within years (Bonferroni corrected for multiple comparisons between years).

| **2018 Vegetation height** | | | |
| --- | --- | --- | --- |
| **Treatments** | **Control** | **Cut** | **Cut + Rotovate** |
| Cut | 0.002 | - | - |
| Cut + Rotovate | <0.0001 | 0.4 | - |
| Rotovate | 0.004 | 1 | 0.09 |
| **2019 Vegetation height** | | | |
| Cut | 0.2 | - | - |
| Cut + Rotovate | 0.02 | 1 | - |
| Rotovate | 0.003 | 1 | 1 |
| **2018 *B. rupestre* cover** | | | |
| Cut | 0.009 | - | - |
| Cut + Rotovate | <0.0001 | 1 | - |
| Rotovate | 0.01 | 1 | 1 |
| **2019 *B. rupestre* cover** | | | |
| Cut | 0.003 | - | - |
| Cut + Rotovate | 0.0002 | 0.01 | - |
| Rotovate | 0.002 | 0.26 | 1 |
| **2021 *B. rupestre* cover** | | | |
| Cut | 0.26 |  |  |
| Cut + Rotovate | 0.07 | 1 | 1 |
| Rotovate | 0.05 | 1 | 1 |
| **2019 Plant species diversity** | | | |
| Cut | 0.3 | - | - |
| Cut + Rotovate | 0.0003 | 0.17 | - |
| Rotovate | 0.008 | 1 | 0.77 |
| **2021 Plant species diversity** | | | |
| Cut | 0.2 | - | - |
| Cut + Rotovate | 0.08 | 1 | - |
| Rotovate | 1 | 1 | 0.48 |

*The table shows data from years where there was a significant difference (p<0.05) between treatments in Kruskal Wallace or Wilcoxon tests. Years with no significant difference between treatment are excluded.*

**Figure S3: a)** Mean summer vegetation height (cm) by year and treatment, measurements were taken in late July/August coinciding with the expected time for female oviposition; b) Spring coefficient of variation in vegetation height to indicate vegetation structure. Boxplots show the median value (horizontal line), upper and lower quartiles (box), the minimum and maximum values (whiskers) and outliers (black circles). Significance values from Kruskal Wallis tests are indicated by *** p=<0.0001, **p=<0.001, *=<0.05, ns = not significant.


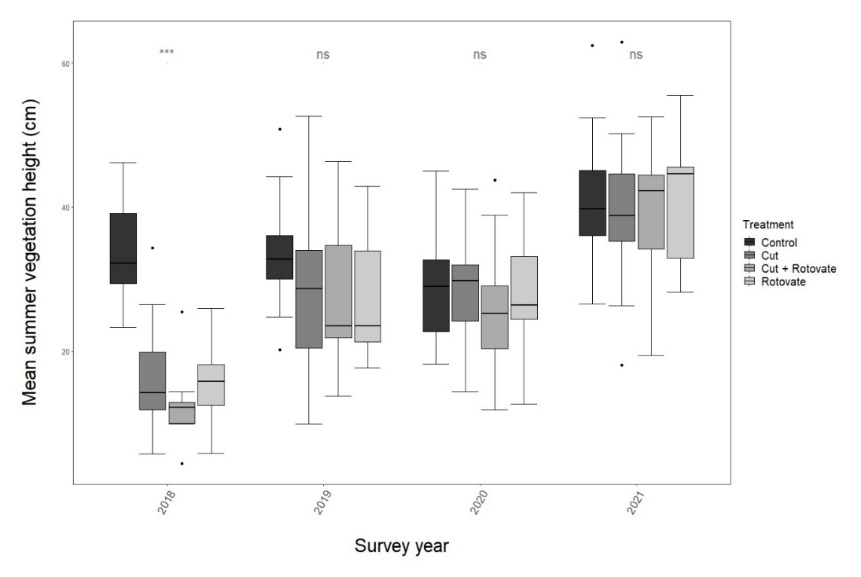
**S3a)**


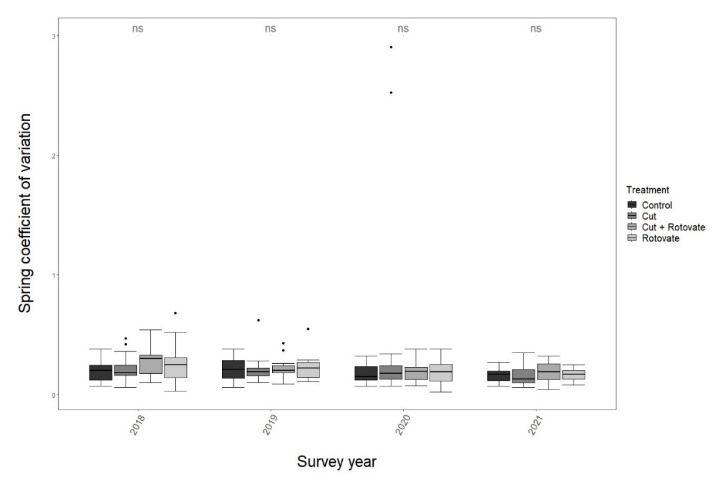
**S3b)**

**Table S4:** Mean vegetation height and *B. rupestre* cover by site and year (bold indicates Wilcoxon test results where there is a significant difference)

| **Habitat attribute** | **Site** | **2017**  **(pre treatment)** | **2018** | **2019** | **2020** | **2021** |
| --- | --- | --- | --- | --- | --- | --- |
| **Average spring vegetation height (cm)** | Dancing Ledge | 39.6 | 20.0 | 20.9 | **34.3** | 29.8 |
|  | Seacombe Cliff | 35.7 | 22.5 | 18.9 | **26.9** | 27.9 |
| **% Tor-grass cover** | Dancing Ledge | 89% | **72%** | 64% | 83% | 83% |
|  | Seacombe Cliff | 84% | **58%** | 59% | 76% | 83% |

*Wilcoxon tests results show significant differences between vegetation height at Dancing Ledge and Seacombe Cliff in 2020 (W = 598, p = 0.002) and in B. rupestre cover in 2018 (W = 551, p = 0.02).*

**Table S5:** Plot occupancy by site, year and treatment. Dancing Ledge has 10 replicates (40 plots), Seacombe Cliff has 5 replicates (20 plots)

| **Treatment** | **Dancing Ledge occupancy by year** | | | | | **Seacombe occupancy by year** | | | | |
| --- | --- | --- | --- | --- | --- | --- | --- | --- | --- | --- |
|  | **2017** | **2018** | **2019** | **2020** | **2021** | **2017** | **2018** | **2019** | **2020** | **2021** |
| **Control** | 3 | 8 | 4 | 4 | 9 | 4 | 2 | 3 | 3 | 5 |
| **Cut** | 2 | 1 | 0 | 4 | 9 | 2 | 0 | 0 | 3 | 2 |
| **Cut + Rotovate** | 2 | 0 | 7 | 7 | 9 | 3 | 1 | 0 | 2 | 2 |
| **Rotovate** | 5 | 3 | 5 | 4 | 8 | 3 | 3 | 2 | 2 | 2 |
| **Total** | **12** | **12** | **16** | **19** | **35** | **12** | **6** | **5** | **10** | **11** |

**Figure S6:** *T. acteon* abundance trend (1992-2021) based on UK Butterfly Monitoring Scheme data. The black box highlights the years during which the management trial were conducted.

**Table S7:** Results from the Generalised Linear Mixed Model with binomial error structure and logit link function and plot as a random effect with larval occupancy (1 = occupied, 0 = unoccupied) again as the dependent variable. Results show relationships between larval occupancy, spring vegetation height (scaled), quadratic effect of spring vegetation height (scaled), host plant cover) treatment and time. There were numerous combinations of models in the compared model set (44) and only models with a delta of <6 are listed along with the null model. The model with the lowest AIC (∆AICc of <2) was used to derive the parameter estimates.

| Model | AICc | ∆AICc | Wi | Intercept | Spring veg | Time | Cut | Rot | Cut x Rot | Cut x time | Rot x time | Cut x rot x time | Prop -ortion *B. rupestre* cover | Spring veg ^2 |
| --- | --- | --- | --- | --- | --- | --- | --- | --- | --- | --- | --- | --- | --- | --- |
| 1 | 220.7 | 0 | 0.691 | -5.652  (1.65) |  | 1.220  (0.474) | -4.248  (2.532) | 3.605  (1.988) | 4.398  (3.104) | 1.054  (0.793) | -1.191  (3.104) | -0.964  (0.982) | 3.349  (1.013) |  |
| 2 | 224.1 | 3.43 | 0.124 | -6.433  (1.894) | -0.265  (0.268) | 1.225  (0.496) | -4.156  (2.517) | 3.652  (2.001) | 4.423  (3.096) | 1.006  (0.793) | -1.209  (3.098) | -0.955  (0.983) | 3.968  (1.235) | 0.026  (0.176) |
| 3 | 226.2 | 5.55 | 0.43 | -4.278  (0.915) |  | 0.723  (0.241) |  | 0.584  (0.387) |  |  |  |  | 2.612  (0.899) |  |
| 4 | 226.4 | 5.69 | 0.04 | -3.839  (0.855) |  | 0.782  (0.241) |  |  |  |  |  |  | 2.181  (0.857) |  |
| 5 | 227.0 | 6.31 | 0.029 | -3.769  (0.943) |  | 0.757  (0.243) | -0.832  (0.516) | -0.097  (0.532) | 1.300  (0.734) |  |  |  | 2.376  (0.901) |  |
| null | 252.3 | 31.67 | 0 | 0.140  (0.164) |  |  |  |  |  |  |  |  |  |  |

*Note*: AICc is the Akaike Information Criterion (corrected for small sample size), ∆AICc the difference in AICc from the best model and Wi is the Akaike weight. The direction of the effect is indicated by model coefficients, with standard errors shown in brackets. Models were fitted using a binomial Generalised Linear Mixed Model with plot as a random intercept.

**Table S8:** Results from a Generalized Linear Mixed Model (GLMM) with a Gaussian error structure, plant species diversity (Simpson’s Index) was the dependent variable, treatment and time were explanatory variables and plot a random effect. Results describe relationships between plant species diversity, treatment and time. The top model with the lowest AICc is used to derive parameter estimates.

| Model | AICc | ∆AICc | Wi | Intercept | Cut | Rotovate | Cut x rotovate | Time | Cut x time | Rotovate x Time | Cut x Rotovate x Time |
| --- | --- | --- | --- | --- | --- | --- | --- | --- | --- | --- | --- |
| 1 | -164.4 | 0 | 0.73 | 0.795  (0.033) | 0.087  (0.029) | 0.082  (0.029) | -0.026  (0.041) | -0.062  (0.009) |  |  |  |
| 2 | -158.6 | 2.84 | 0.17 | 0.867  (0.045) | 0.012  (0.057) |  |  | -0.073  (0.012) | 0.021  (0.017) |  |  |
| 3 | -157.2 | 4.21 | 0.09 | 0.805  (0.040) |  | 0.134  (0.057) |  | -0.051  (0.012) |  | -0.022 (0.017) |  |
| 4 | -140 | 21.41 | 0 | 0.791  (0.056) | 0.028  (0.079) | 0.151  (0.079) | -0.033  (0.112) | -0.061  (0.018) | 0.0197  (0.025) | -0.023  (0.025) | 0.002  (0.035) |
| 5 | -137.9 | 23.51 | 0 | 0.649  (0.016) | 0.074  (0.023) |  |  |  |  |  |  |
| 6 | -136.5 | 24.95 | 0 | 0.652  (0.016) |  | 0.069  (0.023) |  |  |  |  |  |
| Null | -135.7 | 25.70 | 0 | 0.686  (0.012) |  |  |  |  |  |  |  |
| 7 | 133 | 28.41 | 0 | 0.608  (0.022) | 0.087  (0.031) | 0.082  (0.031) | -0.026  (0.045) |  |  |  |  |

*Note*: AICc is the Akaike Information Criterion (corrected for small sample size), ∆AICc the difference in AICc from the best model and Wi is the Akaike weight. The direction of the effect is indicated by model coefficients, with standard errors shown in brackets. Models were fitted using a Generalised Linear Mixed Model with gaussian family and plot as a random intercept.

**Table S9**: List of some identified plant species recorded in 2019 and 2021, tick indicates presence.

| **Plant Species** | **2019** | **2021** |
| --- | --- | --- |
| Agrimony | ✓ |  |
| Autumn hawkbit | ✓ |  |
| Bee Orchid | ✓ |  |
| Bindweed | ✓ | ✓ |
| Bird’s-foot Trefoil | ✓ |  |
| Black Medic | ✓ | ✓ |
| Bristly Ox Tongue | ✓ | ✓ |
| Bulbus Buttercup | ✓ |  |
| Wild Carrot | ✓ | ✓ |
| Clover | ✓ |  |
| Cocksfoot | ✓ | ✓ |
| Common Century | ✓ | ✓ |
| Common Vetch | ✓ |  |
| Creeping Thistle |  | ✓ |
| Cutleaved Cranesbill | ✓ |  |
| Dotted Medic | ✓ |  |
| Dwarf Thistle | ✓ |  |
| Fairy Flax | ✓ |  |
| False Oat Grass | ✓ | ✓ |
| Fescues | ✓ | ✓ |
| Glaucous Sedge | ✓ |  |
| Goose Grass | ✓ | ✓ |
| Greater Knapweed | ✓ | ✓ |
| Hedge Bedstraw | ✓ | ✓ |
| Hoary Plantain |  | ✓ |
| Hop Trefoil | ✓ |  |
| Ivy |  | ✓ |
| Ladies Bedstraw | ✓ | ✓ |
| Mallow |  |  |
| Milkwort | ✓ | ✓ |
| Pale Flax | ✓ |  |
| Parsley Pent | ✓ |  |
| Ploughmans Spikenard | ✓ |  |
| Prickly Sow Thistle | ✓ | ✓ |
| Pyramidal Orchid |  | ✓ |
| Ragwort | ✓ | ✓ |
| Red Valarian |  | ✓ |
| Ribwort Plantain | ✓ | ✓ |
| Rough Hawkbit | ✓ | ✓ |
| Salad Burnet | ✓ | ✓ |
| Smooth Sow Thistle | ✓ |  |
| Spear Thistle | ✓ | ✓ |
| Tare | ✓ | ✓ |
| Teasel |  | ✓ |
| Thyme-leaved Sandwort | ✓ |  |
| Red Valerian | ✓ |  |
| Wild Cabbage | ✓ | ✓ |
| Wild Madder | ✓ | ✓ |
| Wild Onion | ✓ | ✓ |
| Yarrow | ✓ | ✓ |
| Yellow vetch | ✓ | ✓ |
